# *ptf1a^+^*, *ela3l^−^* cells are developmentally maintained progenitors for exocrine regeneration following extreme loss of acinar cells in zebrafish larvae

**DOI:** 10.1242/dmm.026633

**Published:** 2017-03-01

**Authors:** Nicole Schmitner, Kenji Kohno, Dirk Meyer

**Affiliations:** 1 Institute for Molecular Biology, CMBI, University of Innsbruck, 6020 Innsbruck Austria; 2 Graduate School of Biological Sciences, Nara Institute of Science and Technology, 8916-5 Takayama, Ikoma, Nara630-0101, Japan

**Keywords:** Exocrine pancreas, Regeneration, Ablation systems, Wnt pathway, Ptf1a, Zebrafish

## Abstract

The exocrine pancreas displays a significant capacity for regeneration and renewal. In humans and mammalian model systems, the partial loss of exocrine tissue, such as after acute pancreatitis or partial pancreatectomy induces rapid recovery via expansion of surviving acinar cells. In mouse it was further found that an almost complete removal of acinar cells initiates regeneration from a currently not well-defined progenitor pool. Here, we used the zebrafish as an alternative model to study cellular mechanisms of exocrine regeneration following an almost complete removal of acinar cells. We introduced and validated two novel transgenic approaches for genetically encoded conditional cell ablation in the zebrafish, either by caspase-8-induced apoptosis or by rendering cells sensitive to diphtheria toxin. By using the *ela3l* promoter for exocrine-specific expression, we show that both approaches allowed cell-type-specific removal of >95% of acinar tissue in larval and adult zebrafish without causing any signs of unspecific side effects. We find that zebrafish larvae are able to recover from a virtually complete acinar tissue ablation within 2 weeks. Using short-term lineage-tracing experiments and EdU incorporation assays, we exclude duct-associated Notch-responsive cells as the source of regeneration. Rather, a rare population of slowly dividing *ela3l-*negative cells expressing *ptf1a* and CPA was identified as the origin of the newly forming exocrine cells. Cells are actively maintained, as revealed by a constant number of these cells at different larval stages and after repeated cell ablation. These cells establish *ela3l* expression about 4-6 days after ablation without signs of increased proliferation in between. With onset of *ela3l* expression, cells initiate rapid proliferation, leading to fast expansion of the *ela3l*-positive population. Finally, we show that this proliferation is blocked by overexpression of the Wnt-signaling antagonist *dkk1b*. In conclusion, we show a conserved requirement for Wnt signaling in exocrine tissue expansion and reveal a potential novel progenitor or stem cell population as a source for exocrine neogenesis after complete loss of acinar cells.

## Abstract (precis)

**Summary:** New transgenic models for exocrine pancreas regeneration in zebrafish reveal a novel potential exocrine progenitor as the source for a two-phase regeneration process after complete acinar cell loss.

## INTRODUCTION

The pancreas is a vertebrate-specific endodermal organ executing major functions in food digestion and glucose homeostasis. The mature organ is composed of an exocrine compartment with acinar and duct cells that produce and transport digestive enzymes into the gut, and an endocrine compartment from which metabolism-regulating peptide hormones including insulin are secreted into the blood stream. Studies on pancreas regeneration have focused mainly on the endocrine compartment, with the aim of gaining knowledge on β-cell regeneration. More recently, exocrine pancreas regeneration has been receiving more attention in the context of cancer and diabetes research because experimental models revealed a high capacity for cell fate plasticity (Murtaugh and Keefe, 2015).

Regeneration of exocrine tissue has been mainly studied in mouse models, where the loss of cells can be induced by caerulein treatment or partial duct ligation (Aghdassi et al., 2011; Lerch and Gorelick, 2013). Using these procedures, repair mechanisms such as acinar cell proliferation along with de-differentiation and re-differentiation processes were described (Desai et al., 2007; Strobel et al., 2007). Because of the lack of lineage-tracing experiments, the origin of regenerating acinar cells could not be determined. Possible sources include acinar cells, centroacinar cells, duct cells or adult progenitor cells (Siveke et al., 2008). The latest findings suggest that more extreme acinar loss in *Ela^Cre-ERT2^;R26^DTR^* mice is not only repaired by acinar cell proliferation but also by differentiation of non-acinar cells (Criscimanna et al., 2011). The nature of these cells has not been clarified, although the data suggest involvement of duct or duct-associated cells.

Here, we used the zebrafish as an alternative model for studying exocrine pancreas regeneration. Importantly, the pancreata in mammalian systems and fish have not only conserved physiological functions and similar cellular compositions and structures, but also conserved expression and function of most genes involved in organ development (Argenton et al., 1999; Biemar et al., 2001; Eames et al., 2010; Jurczyk et al., 2011; Lin et al., 2004; Yee et al., 2005; Zecchin et al., 2004). Similar to mammals, the zebrafish pancreas arises from two progenitor domains called the dorsal bud and the ventral bud (Field et al., 2003; Hesselson et al., 2009). The dorsal bud develops after 24 hours post fertilization (hpf) and consists exclusively of clustered early endocrine cells known as the principal islet. The ventral bud appears after 36 hpf, grows to engulf the principal islet (Field et al., 2003; Wang et al., 2011) and gives rise to later-forming endocrine cells and all exocrine compartments (Field et al., 2003; Hesselson et al., 2009; Lin et al., 2004; Wang et al., 2011; Zecchin et al., 2004). Development of the exocrine pancreas, as described by Yee et al. (2005) using histological, immunohistochemical and ultrastructural approaches, can be followed by the successive induction of *trypsin* (48 hpf), carboxypeptidase A (CPA) (60 hpf) and *elastase 3 like* (*ela3l*). However, the molecular cascades of events involved in exocrine pancreatic differentiation and regeneration in zebrafish are not well defined.

As part of this study, we also introduced two novel approaches for genetically induced cell ablation into the zebrafish. One method uses the application of diphtheria toxin (DT) in combination with transgene-driven, exocrine-cell-specific expression of the human DT receptor (HB-EGF), rendering cells sensitive to DT (Furukawa et al., 2006; Saito et al., 2001). In the second approach, cell-type-specific apoptosis can be induced by conditional dimerization of a membrane-bound caspase-8-FKBP fusion protein, which then initiates the caspase cascade. Both techniques have been successfully established in mouse models to ablate β-cells of the endocrine pancreas, exocrine cells and adipocytes (Criscimanna et al., 2011; Pajvani et al., 2005; Thorel et al., 2010), but have not previously been applied in zebrafish.

By introducing these systems into the pancreas of zebrafish, we were able to efficiently ablate acinar cells in larval through to adult stage animals, and we proceeded with a detailed examination of the exocrine regeneration process. Studies on larval pancreas regeneration revealed different mechanisms and dynamics of regeneration depending on the time point of treatment. While our studies found no evidence for an involvement of duct and centroacinar cells in exocrine regeneration, they revealed the presence of a previously uncharacterized *ptf1a^+^*, *ela3l^−^* cell population and propose that these cells are a novel type of pancreatic progenitor cell. Following a virtually complete removal of acinar gland cells, these *ptf1a^+^*, *ela3l^−^* cells differentiated into exocrine cells and restored exocrine cell mass by subsequent Wnt-signaling-dependent proliferation.

## RESULTS

### Complete exocrine cell ablation using two novel cell-ablation approaches in zebrafish larvae and adults

**Fig. 1.**
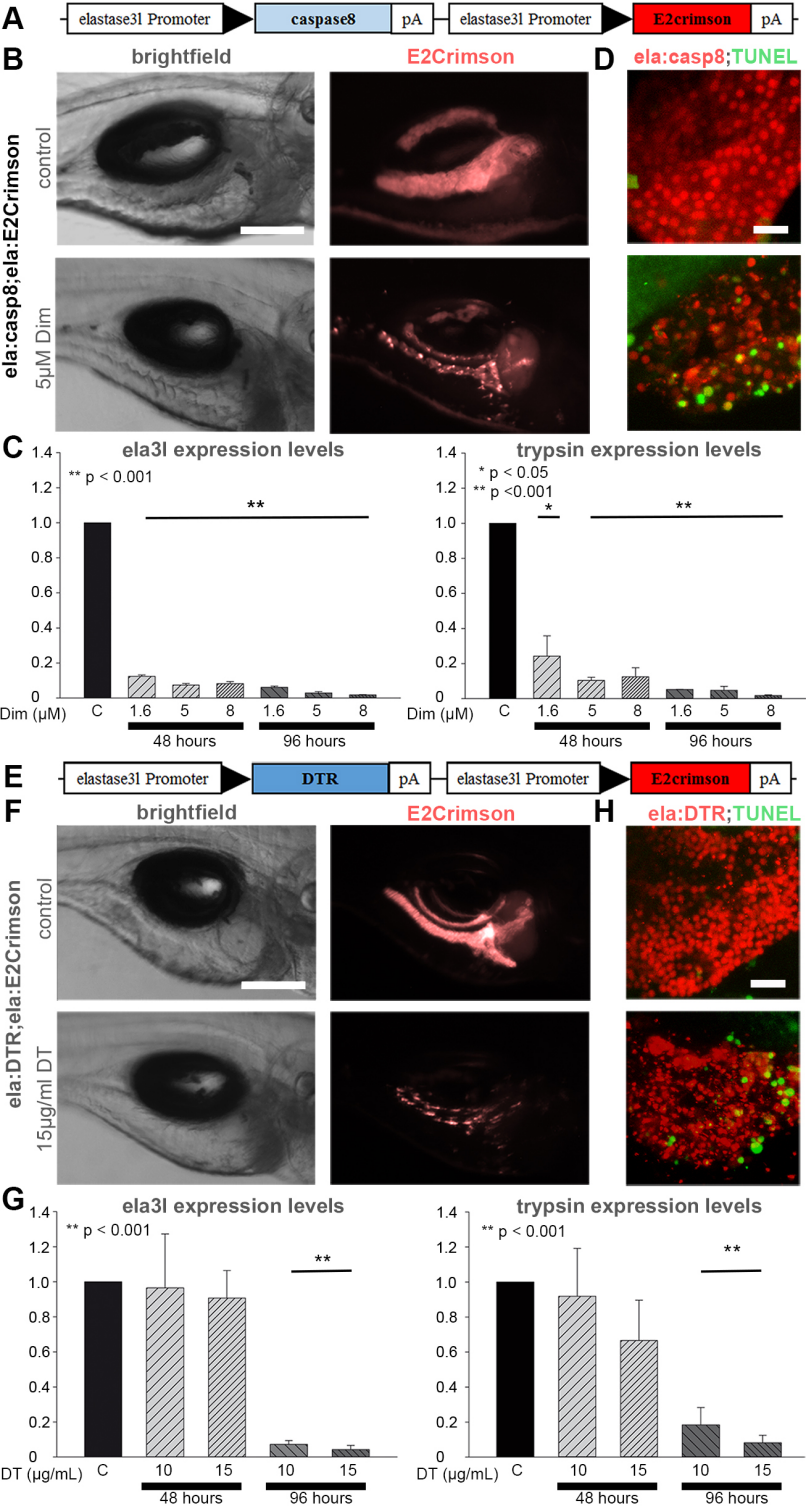
**Exocrine-tissue-specific zebrafish cell ablation systems.** (A) Construct used to generate *ela:casp8* transgenics (black arrowheads indicate promoter direction). (B) Brightfield and epifluorescence images of 7 dpf larvae showing E2Crimson signals in untreated control animals and after ablation with 5 µM Dim for 48 h. (C) Relative expression levels of *ela3l* (left) and *trypsin* (right) mRNA as revealed by qPCR analyses of whole-embryo RNA preparations of control larvae and larvae treated with 1.6, 5 and 8 µM Dim for 48 and 96 h, normalized to *β-actin* levels. (D) Confocal projection of untreated 5 dpf larva and larva treated for 8 h with 5 µM Dim labelled by TUNEL staining (green). (E) Construct used to generate the *ela:DTR* transgenics. (F) Brightfield and epifluorescence images of 9 dpf untreated larvae showing exocrine-specific expression of E2Crimson and strongly reduced expression of E2Crimson after incubation in 15 µg/ml DT for 96 h. (G) Relative expression levels of *ela3l* (left) and *trypsin* (right) mRNA as revealed by qPCR analyses of whole-embryo RNA preparations of control larvae and larvae treated with 10 and 15 µg/ml DT for 48 and 96 h, normalized to *β-actin* levels. (H) Confocal projection of untreated 7 dpf larva and larva treated for 72 h with 15 µg/ml DT labeled by TUNEL staining (green). Values are mean+s.e.m. of *n*=5 embryos per sample and >3 biological replicates. Scale bars: 150 µm (B,F) and 20 µm (D,H).

Currently, the most efficient system for genetically encoded conditional cell ablation in zebrafish is based on the transgenic expression of bacterial nitroreductase (NTR) to sensitize specific cells to the antibiotic metronidazole (Met; Curado et al., 2007; Pisharath et al., 2007). The possibility of unwanted side effects from metronidazole interfering with microbiota led us to explore two alternative ablation methods, which had been shown to enable almost complete removal of pancreatic and other cell types in adult mice. These approaches either utilize induction of apoptosis through caspase 8 or they render cells sensitive to diphtheria toxin (Carlotti et al., 2005; Criscimanna et al., 2011; Pajvani et al., 2005; Thorel et al., 2010). To test these techniques in the context of exocrine pancreas regeneration, we generated transgenic lines expressing the mediator proteins together with the *in vivo* reporter E2Crimson under control of the acinar-specific *ela3l* promoter (Wan et al., 2006). In Tg(*ela3l:casp8;ela3l:E2Crimson*) embryos (termed *ela:casp8*) the mediator protein consists of the p20 and p10 catalytic domains of human caspase 8 fused to serial FKBPv dimerization domains and a myristoylation site to provide membrane attachment (Pajvani et al., 2005) (Fig. 1A). Incubation of 5 dpf *ela:casp8* embryos in 5 µM dimer-inducing agent AP20187 (termed Dim) resulted in loss of E2Crimson signal within 2 days of treatment (0 days post ablation or 0 dpa) (Fig. 1B). To determine optimal conditions for ablation and to test for potential side effects, embryos were treated with 3 different concentrations of Dim (1.6 µM, 5 µM and 8 µM) for two different time periods (48 and 96 h). Quantification of *ela3l* and *trypsin* mRNA expression levels via RT-qPCR confirmed a dose- and time-dependent removal of exocrine tissue (Fig. 1C). The lowest concentration of 1.6 µM Dim resulted in 85% and 77% reduced *ela3l* and *trypsin* levels after 48 h of treatment and a 95% reduction of these RNAs after 96 h. Treatment with 8 µM Dim caused an *ela3l* and *trypsin* reduction of 95% and 94% after 48 h and 98% to 99% (96 h), respectively. Importantly, the prolonged treatment for 96 h did not cause any obvious side effects as revealed by careful macroscopic analyses of more than 100 embryos and larvae (data not shown). Caspase-8-induced apoptosis was detected in exocrine cells as early as 8 h after administration of Dim, as revealed by TUNEL assays (Fig. 1D). Time-lapse analysis of an *ela:casp8* embryo treated with 5 µM Dim recorded between 6 and 16 h after the start of treatment visualized the accumulation of E2Crimson and the concurrent loss of E2Crimson-expressing cells (Movie 1). Apoptotic ablation of exocrine tissue was confirmed by the loss of cell membrane integrity in *cldn:lyn-eGFP* embryos (Haas and Gilmour, 2006), in which all epithelial cells are marked by membrane-tagged eGFP (Fig. S1A,B). Based on these results, treatment with 5 µM Dim for 48 h was used for further experiments.

The second ablation system uses the human diphtheria toxin receptor HB-EGF (Furukawa et al., 2006) (DTR) to make cells sensitive to administrated diphtheria toxin (DT) (Fig. 1E). Neither the expression of human DTR in Tg(*ela3l:DTR;ela3l:E2Crimson*) (termed *ela:DTR*) animals nor the incubation of wild-type animals in DT concentrations up to 50 µg/ml caused any distinguishable phenotypes in more than 100 analyzed control embryos and larvae (data not shown). However, incubation of *ela:DTR* transgenic fish in 10 µg/ml and 15 µg/ml DT caused reduced E2Crimson signals at 72 h of treatment. Moreover, an almost complete loss was achieved after 96 h (Fig. 1F). RT-qPCR analyses showed that expression of exocrine markers was unchanged after 48 h of treatment with 15 µg/ml DT, while after 96 h of treatment, *ela3l* mRNA was reduced by 93% (10 µg/ml DT) and 96% (15 µg/ml DT), and *trypsin* levels were reduced by up to 92% (Fig. 1G). Further analyses of DT-treated *ela:DTR* animals by TUNEL and in a *cldn:lyn-eGFP* background (Fig. 1H; Fig. S1C) revealed the first apoptotic cells after 72 h of treatment. The data show that both ablation approaches allow an almost complete removal of larval exocrine tissue within 4 days of treatment and that kinetics of apoptosis induction were slightly slower in DT-treated *ela:DTR* fish compared with Dim-treated *ela:casp8* animals.

**Fig. 2.**
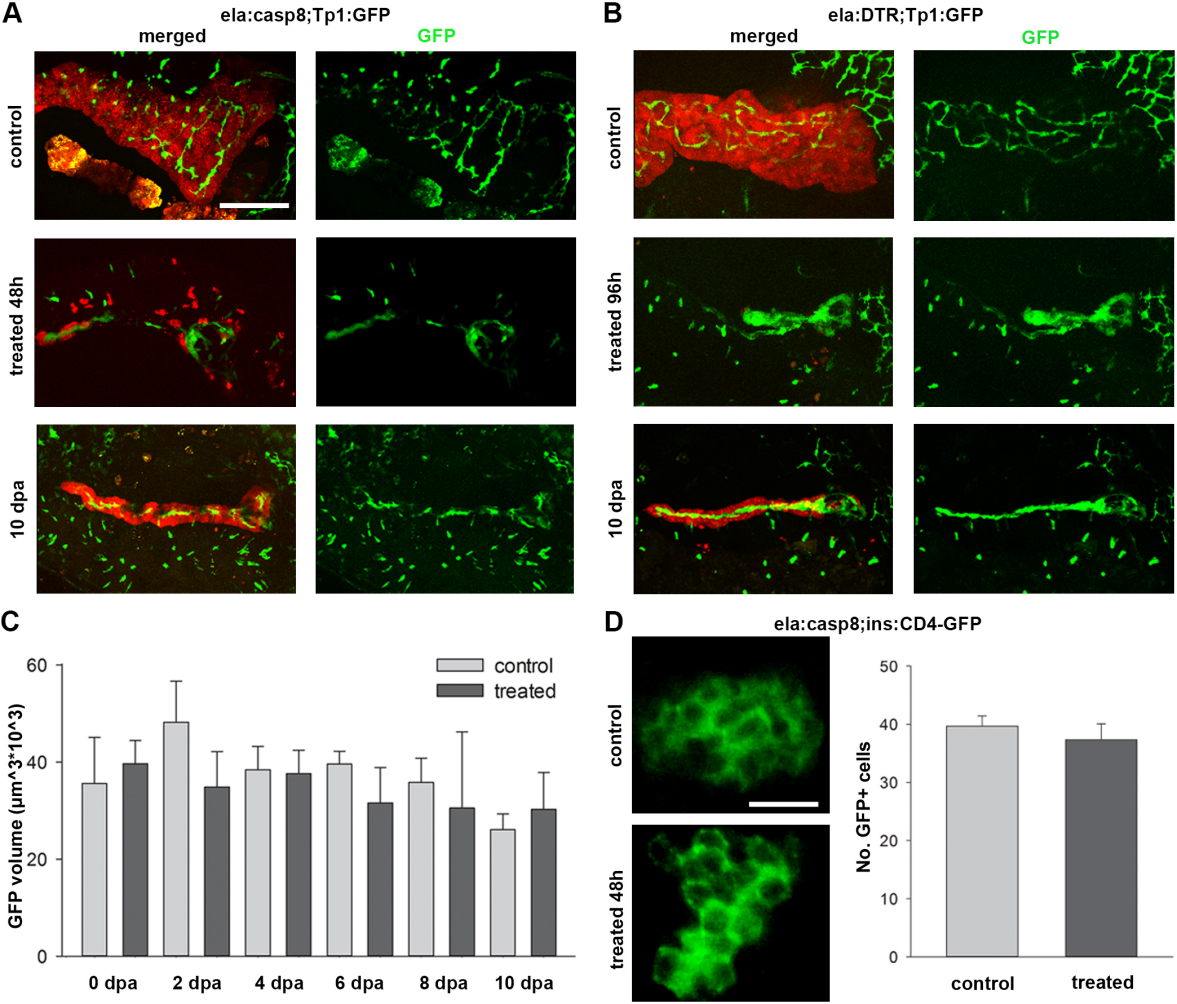
**Ablation of exocrine cells does not affect neighboring NRCs and β-cells.** (A,B) Confocal image projections of *ela:casp8;Tp1:eGFP* (A) and *ela:DTR;Tp1:eGFP* (B) in untreated control animals (7 dpf for *ela:casp8* and 9 dpf for *ela:DTR*), animals directly after treatment and 10 dpf animals. Note that newly established exocrine cells are found next to NRCs. (C) Volume of pancreatic NRCs, determined by GFP expression, is not significantly changed after ablation and during regeneration of exocrine cells in *ela:casp8* embryos (*n*>5 larva for each time point). (D) β-cells labeled using Tg(*ins:CD4-eGFP*). The number of β-cells was unaffected by treatment with dimerizer (*n*=6). Values are mean+s.e.m. Scale bars: 100 µm (A,B) and 20 µm (D).

To determine if neighboring cells are affected by the ablation of exocrine cells, treatment using either *ela:casp8* (with 5 µM Dim) or *ela:DTR* transgenes (with 15 µg/ml DT) were performed in GFP-reporter backgrounds for Notch-responsive cells (NRCs) (*Tp1:GFP*; Fig. 2A-C) and β-cells (*ins:CD4-GFP*; Fig. 2D). Notably, pancreatic NRCs have recently been revealed to be centroacinar cells serving as endocrine progenitors (Beer et al., 2016; Delaspre et al., 2015; Ghaye et al., 2015). Removal of exocrine cells in the *Tp1:eGFP* background resulted in the disruption of the net-like organization of the NRCs. However, pancreas-specific GFP volumes in these fish were not affected at any time point after treatment, suggesting that the total number of NRCs was unaffected by the treatment (Fig. 2C). Using a transgenic line labeling β-cells via eGFP fused to the membrane-localized human CD4 protein, we also found that neither the islet morphology nor the number of β-cells was affected by acinar cell ablation (Fig. 2D).

As the examination of repair and regeneration, especially in adult stages, is becoming more important, we tested the ability of our alternative ablation methods to remove exocrine cells in >6-month-old adult fish carrying either the *ela:casp8* or the *ela:DTR* transgene. In *ela:casp8* animals, a single injection of 20-30 ng/g Dim achieved a nearly complete ablation after 14 days (*n*=5, data not shown), whereas the 75 ng/g Dim injection caused the loss of fluorescent signals within 4 days (Fig. S2A). Adult *ela:DTR* fish were injected once with 20 ng/g DT intraperitoneally and subsequently displayed strongly reduced fluorescence 3-4 days post injection (Fig. S2B). The removal of exocrine cells was confirmed in histological sections of inner organs (Fig. S2A′,B′). In addition, we established transgenic lines for the ablation of the insulin-producing β-cells (Fig. S2C,D). Both treatments led to the loss of E2Crimson-expressing β-cells (97% for the capase-8-induced ablation and 96% for the DT-mediated ablation) and correspondingly reduced relative expression of insulin mRNA (96% for the capase-8-induced and 91% for the DT-mediated ablation) (Fig. S2E). In conclusion, these studies demonstrate that both the inducible activation of caspase 8, as well as the expression of human DTR, enable efficient cell-type-specific ablation in embryonic and adult stages in zebrafish.

### Dynamics of exocrine cell regeneration

**Fig. 3.**
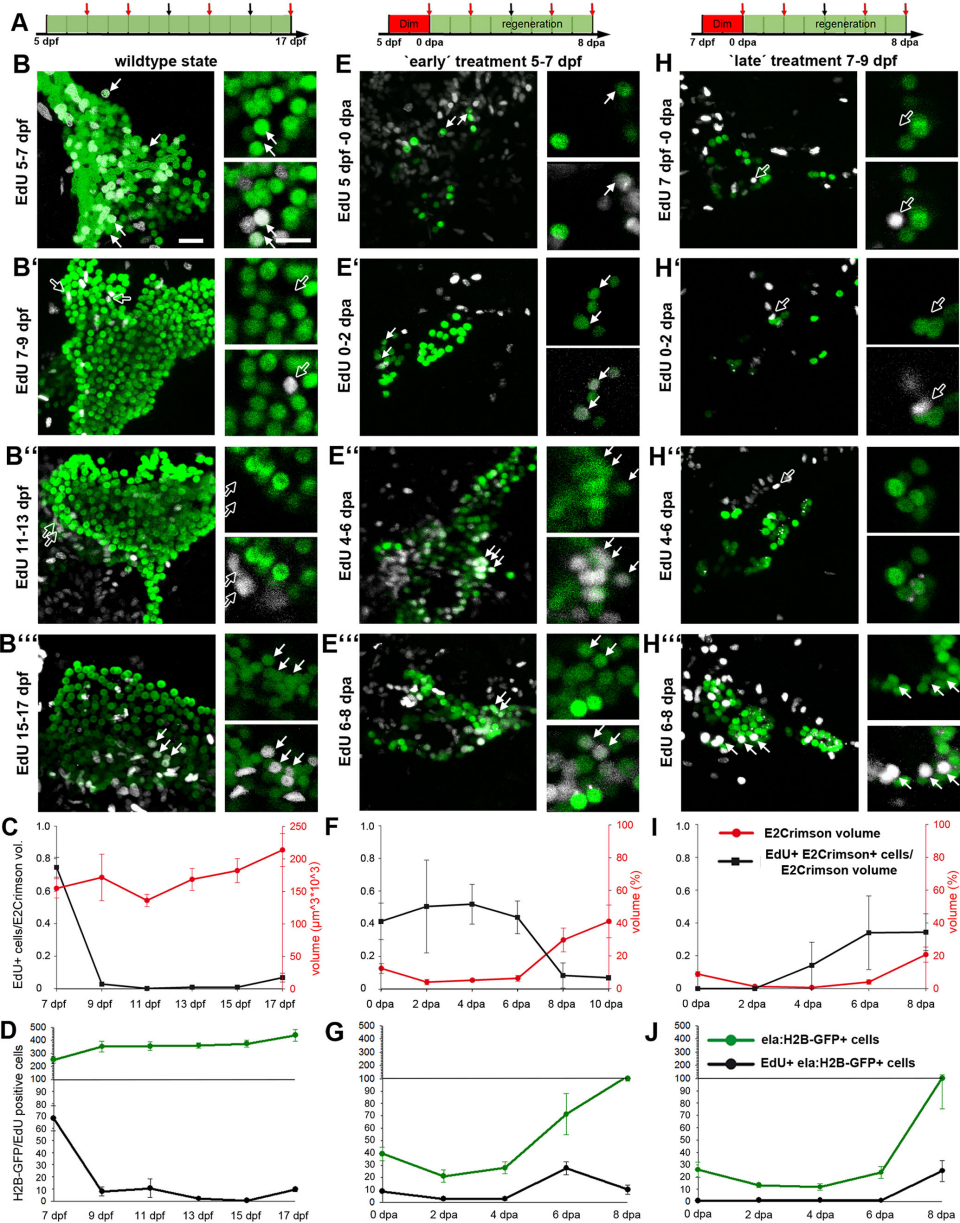
**Dynamics of exocrine development and regeneration.** (A) Time scheme of regeneration experiments indicating phase with Dim treatment (in red) and time points of fixation (arrows). (B,E,H) Confocal images of Tg(*ela:casp8;ela:H2B-GFP*) animals at different stages of larval development (stages as indicated by red arrows in A). All larvae were treated with EdU for 48 h before fixation (GFP signals in green, EdU signals in white, smaller white dots correspond to E2Crimson background). Images cover the pancreatic head region of untreated (B-B′″) animals and of larvae treated with 5 µM Dim either from 5 to 7 dpf (E-E″′, ‘early’ treatment) or from 7 to 9 dpf (H-H′″, ‘late’ treatment). Shown are projections and selections of single plane images (smaller images to the right). Note that ‘late’ treatment larva between 0-6 dpa lack EdU^+^ GFP^+^ cells (white arrows) and that EdU^+^ signals in 9 dpf and 13 dpf control animals (B′,B″) and in 0-6 dpa late treatment larvae (H-H″) localized to GFP-negative nuclei (black arrows). (C,D,F,G,I,J) Data quantification (*n*>5 larva for each time point; mean+s.e.m.) using volumetric measurements (C,F,I) and cell counts (D,G,J: absolute numbers of nuclei per stack) for control larva (C,D), early treatment larvae (F,G) and late treatment larvae (I,J). Red line in C shows the absolute E2Crimson volume (in red), whereas in F and I, it shows the relative volume compared with the wild-type situation as shown in C. Note that different samples were analyzed in E-G and H-J, and that the higher proliferation rate in 11 dpf control animals shown in D compared with C result from only two larvae with 10 and 40 EdU^+^ cells (see Table S1). Scale bars: 20 µm.

The analyses of *ela:casp8;Tp1:eGFP* and *ela:DTR;Tp1:eGFP* larvae at 10 dpa revealed that both lines had re-established E2Crimson-positive cells directly adjacent to the GFP-positive NRCs (Fig. 2A,B). For a more detailed understanding of the regeneration dynamics, *ela:casp8* embryos that had been treated with Dim from 5 to 7 dpf were analyzed at 2 day intervals from 0 to 8 dpa (Fig. 3A-G). In addition, EdU treatments starting 2 days before fixation were used to correlate proliferation behavior with regeneration dynamics (Hesselson et al., 2009; Kramer-Zucker et al., 2005). Fixed and stained larvae were documented by 3D confocal microscopy (Fig. 3B,E). As clumping of E2Crimson protein prevented accurate cell assignments, a volumetric approach was used for quantification of E2Crimson staining (Fig. 3C,F; Fig. S3). In addition, experiments were performed in ela3l:casp8/*ela3l:H2B-GFP* double transgenic animals to enable cell-counting based on nuclear-specific H2B-GFP expression (Fig. 3B,D,E,G; Table S1). Consistent with the RT-qPCR analyses, volumetric analyses (Fig. 3C,F) and counting of cells (Fig. 3D,G) showed a 94% reduction of the ela3l^+^ cells at 2 dpa. Between 2 and 4 dpa, ela3l^+^ cell mass increased to 8% of the control larvae, while between 6 and 8 dpa a rapid increase of exocrine tissue occurred reaching 18% and 26% of control levels, respectively. Unlike control embryos, which mainly lacked EdU^+^, *ela3l*^+^ cells between 9 and 17 dpf (Fig. 3B-D), Dim-treated embryos showed elevated proliferation rates at 6 dpa and lower rates at 8 dpa (Fig. 3E-G). The data imply proliferation of the remaining E2Crimson^+^ cells as a mechanism of exocrine tissue repair. We speculated that these cells were either resistant to Dim or they may have escaped ablation because of a later onset of caspase-8/E2Crimson expression only after Dim exposure. We considered resistance unlikely as the reduction of E2Crimson^+^ tissue volume between 0 dpa (16% of control levels) and 2 dpa (6%) suggests that eventually all *ela:casp8*-expressing cells undergo apoptosis, but with slightly variable dynamics. Based on the high proliferation rate of *ela3l* cells seen between 5 and 7 dpf in control embryos (Fig. 3B-D), we hypothesized that some cells were not fully matured and therefore did not express *ela3l* during the treatment. In this case, a treatment starting at 7 dpf, which is after the drop in proliferation, should result in a more complete removal of exocrine tissue. To better distinguish between an ablation-induced regeneration response and the normal development based exocrine differentiation, regeneration experiments and 2 day EdU treatments were repeated in *ela:casp8* larvae treated with Dim at 7-9 dpf (Fig. 3H-J). Consistent with our expectation, this ‘late’ treatment resulted in a virtually complete loss of ela3l^+^ cells at 2 and 4 dpa (Fig. 3I,J). This was confirmed by RT-qPCR analyses, which revealed 2.6-times lower *ela3l* mRNA levels after 7-9 dpf Dim treatment compared with ‘early’ treatments (Fig. S4). Unlike the situation in early-treated larvae, very few proliferating E2Crimson^+^ cells were found before 6 dpa (Fig. 3I,J′). However, at 8 dpa, rapidly increasing numbers of ela3l^+^ cells were observed in late-treated larvae and at 8 dpa, these larvae had recovered about 23% tissue compared with control animals (Fig. 3H′″,I,J).

While exocrine tissue repair in larvae ablated at 5-7 dpf may happen through self-replication of late-maturing *ela3l*^+^ cells, the data for the ‘late’ treatment suggest the existence of an alternative regeneration path using ela^+^ cell neogenesis.

### Regeneration of exocrine cells does not involve ductal cells

Studies on exocrine differentiation in mouse showed different modes of regeneration. A partial loss of exocrine tissue appears to be compensated mainly by the proliferation of remaining exocrine cells (Desai et al., 2007; Strobel et al., 2007), whereas complete removal induces exocrine neogenesis from a currently undefined progenitor population (Criscimanna et al., 2011). Pancreatic duct cells had been suggested as a potential source of this progenitor population, even though lineage-tracing studies supporting this hypothesis are lacking (Criscimanna et al., 2011).

**Fig. 4.**
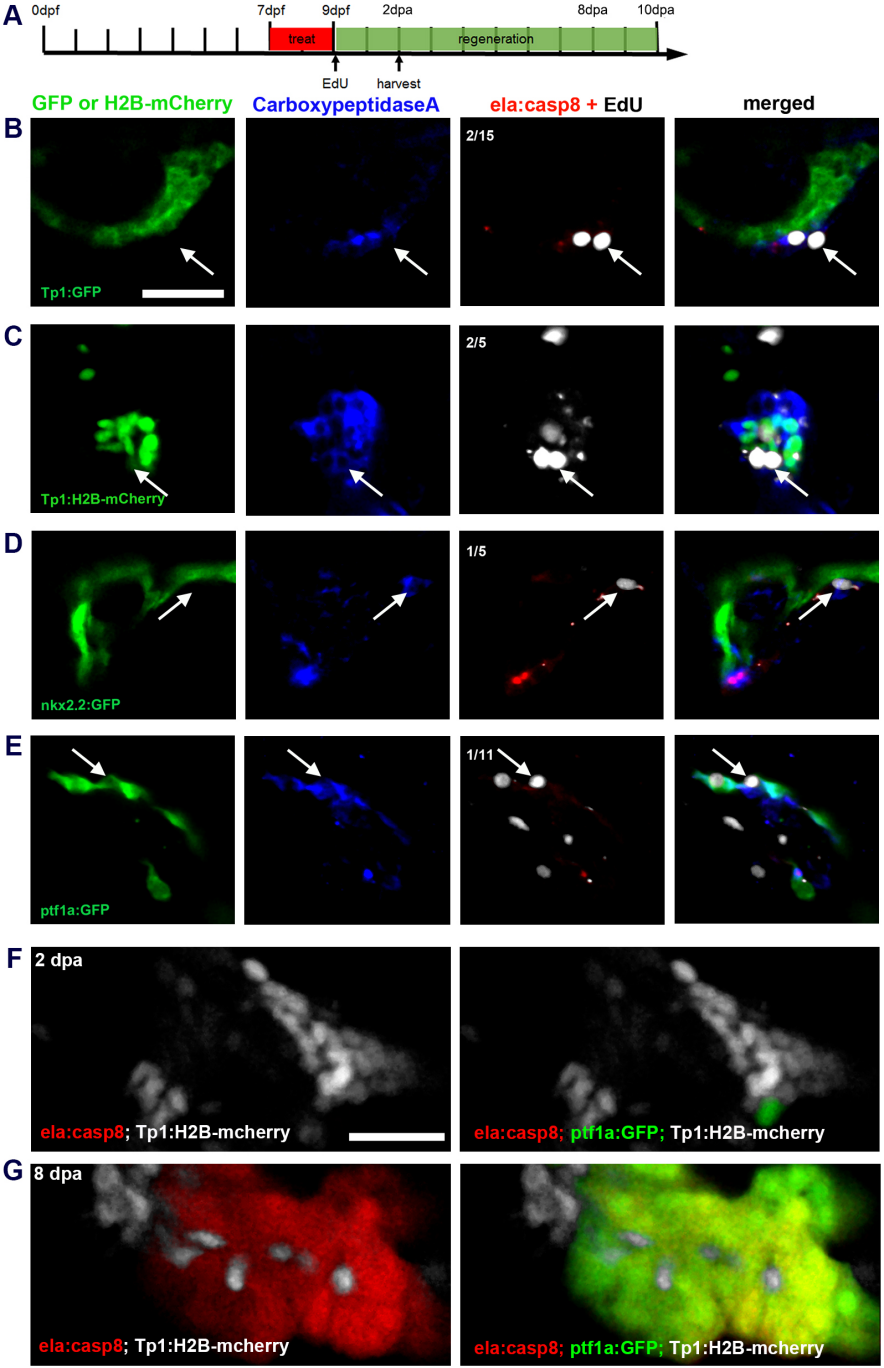
**Identification of progenitor cells in the regenerating exocrine pancreas.** (A) Timeline of short-term regeneration experiments shown in B-E indicating the time window of Dim treatment (red, 7-9 dpf), starting point of EdU treatment (0 dpa) and the fixation time (2 dpa). (B-E) Confocal image projection showing different lineage reporters (green), CPA antibody signals (blue), EdU signals (white, arrows indicate proliferating CPA^+^ cells) and E2Crimson (red; in C, E2Crimson and EdU staining are both shown white due to the *Tp1:H2B-mcherry* background). Note that CPA^+^ cells are negative for duct/NRC reporter expression in *Tp1:eGFP* (B), *Tp1:H2B-mcherry* (C) and *nkx2.2:eGFP* (D), whereas CPA signals match expression of *ptf1a:GFP* (E). (F,G) Comparison of reporter expression for NRCs (Tp1:H2B-mcherry, white), Ptf1a^+^(ptf1a:eGFP; green) and ela3l^+^ (*ela:casp8*, red) in triple transgenic larva at 2 dpa (F) and 8 dpa (G). NRCs signals localize next to Ptf1a^+^ cells at 2 dpa (F) and they intermingle with Ptf1a^+^ and ela3l^+^ labels at 8 dpa (G), but they do overlap with these markers. Scale bars: 20 µm.

To clarify whether duct cells contribute to exocrine tissue regeneration in zebrafish, we performed short-term tracing experiments with the duct-specific reporter lines Tg(*Tp1:eGFP*), Tg(*Tp1:H2B-mCherry*) and Tg(*nkx2.2a:eGFP*) (Ninov et al., 2012; Parsons et al., 2009; Pauls et al., 2007). In these experiments, *ela:casp8* larvae, treated with Dim from 7 to 9 dpf were analyzed at different time points during regeneration. We found no evidence for expression of NRCs or duct cell reporters in newly forming exocrine cells at 8 dpa (Fig. S5). A loss of lineage tracer by protein decay is unlikely as H2B-mCherry had been reported to be stable for weeks (Brennand et al., 2007; Hesselson et al., 2009). To exclude the dilution of lineage trace by massive proliferation, we also performed 2 day EdU treatment starting directly after Dim treatment (Fig. 4, Fig. S6). We found that the majority of eGFP/mCherry-positive cells in 2 dpa larvae had no EdU signal. Hence, we concluded that neither Notch-responsive cells nor other duct cells are a source for newly established exocrine cells.

### Identification of *ptf1a*^+^, CPA^+^, *ela3l*^−^ cells during exocrine regeneration

In order to identify regenerating cells at an earlier stage of maturation, we performed antibody staining for the early maturation marker CPA (Zhou et al., 2007). Analyses at 2 dpa revealed rare CPA^+^, E2Crimson^−^ cells (5-8 cells per embryo) next to the eGFP- or H2B-mCherry-labeled duct cells (Fig. 4). Few of these CPA^+^ cells contained EdU label, indicating a low proliferation rate (in total 6 cells in 36 embryos; Fig. 4B-D). Therefore, we hypothesized that CPA staining marks slowly proliferating progenitors of the regenerating exocrine cells. As pancreas-specific transcription factor 1a (Ptf1a) is essential for differentiation of exocrine pancreas (Dong et al., 2008; Lin et al., 2004; Zecchin et al., 2004), we expected a regulation of *ptf1a* in such progenitors cells at some stage of exocrine regeneration. To correlate CPA expression with that of *pft1a* during pancreatic regeneration, we performed ablation studies combined with EdU incorporation experiments in the *ptf1a:eGFP* background. We found that Dim treatment did not remove all *ptf1a:eGFP*^+^ cells and that CPA expression and the rare EdU labels were restricted to the pool of these remaining GFP^+^ cells (Fig. 4E). Genetic lineage-tracing studies previously showed that Notch-responsiveness and *ptf1a* expression correlates with mainly different pancreatic lineages (Wang et al., 2011, 2015). Consistently, no overlap for GFP and mCherry was found in *ptf1a:eGFP*/*Tp1:H2B-mCherry* and *ela3l:H2B-GFP*/*Tp1:H2B-mCherry* control animals between 3 and 7 dpf (Fig. S7). By investigating 2 dpa larvae in triple transgenic *ela:casp8;ptf1a:eGFP;Tp1:H2B-mCherry* animals, we further found that *ptf1a^+^*, E2Crimson^−^ cells reside next to NRCs at 2 dpa and 8 dpa but that they lack the NRC-lineage marker H2B-mCherry and therefore are also not derived from NRCs (Fig. 4F).

### Presence of *ptf1a*^+^, *ela3l*^−^ cells during normal development and exocrine regeneration

**Fig. 5.**
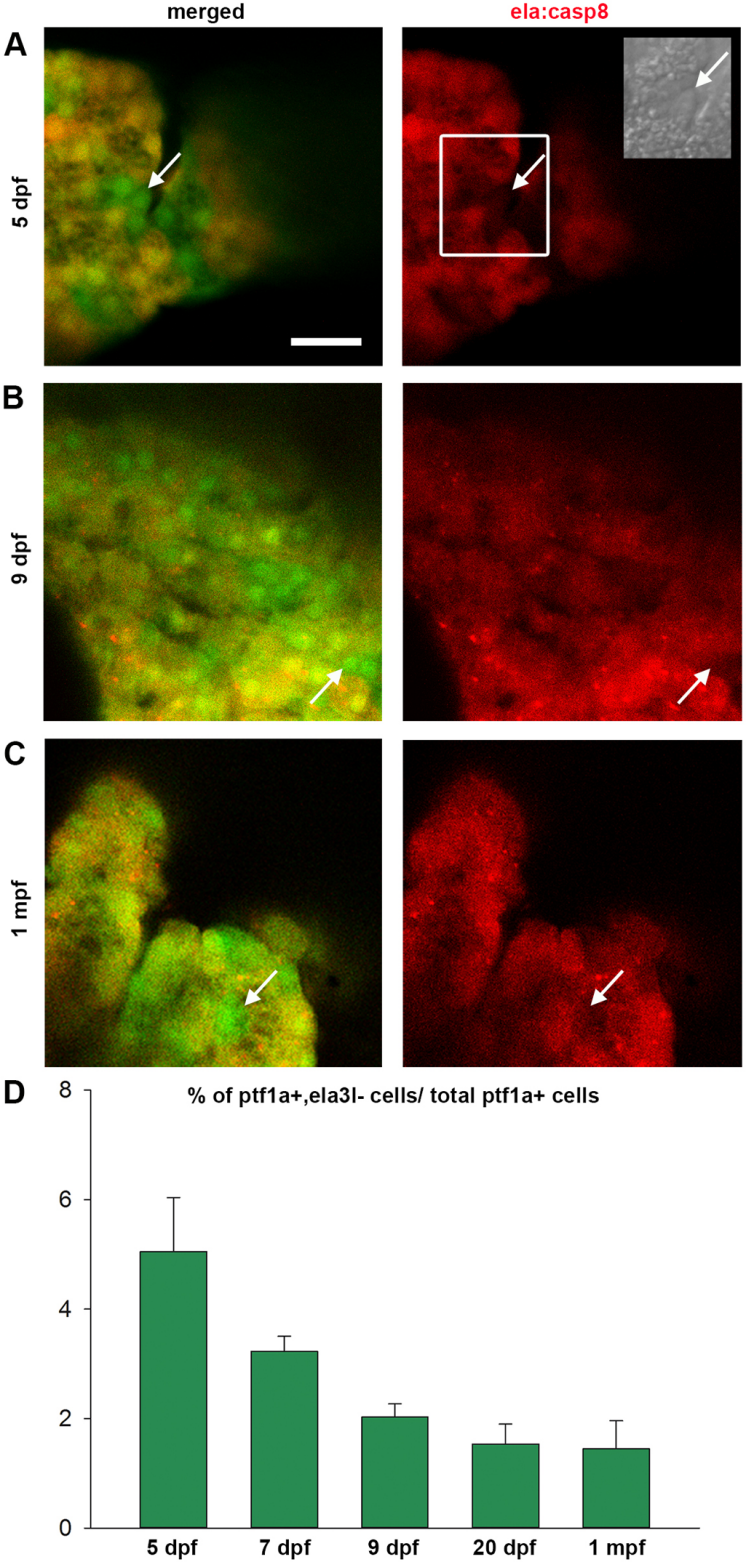
**Ptf1a^+^, ela3l^−^ cells are present at different developmental stages.** (A-C) Confocal images of the anterior pancreatic region of Tg(*ela:casp8; ptf1a:eGFP*) in 5 dpf (A), 9 dpf (B) and 30 dpf (C) animals (arrows indicate selected GFP^+^, e2Crimson*^−^* cells). (D) Relative amount of *ptf1a^+^*, *ela3l^−^* cells at different developmental stages (*n*>5). Mean+s.e.m. Scale bar: 20 µm.

Detailed analyses of confocal images taken from *ptf1a:eGFP;ela:casp8* animals at different stages of development showed GFP^+^, E2Crimson^−^ cells, not only in early larval stages, but also in juvenile fish (Fig. 5A-C). Corresponding cells preferentially localized to the pancreas periphery where they were found intermingled with E2Crimson^+^ cells. Manual examination of confocal image stacks taken from pancreatic head of regions of 5 dpf to 30 dpf animals was used to estimate their frequency of occurrence. The studies also revealed that eGFP^+^, E2Crimson^−^ cells lacked enzyme granules seen in all E2Crimson^+^ cells, providing an additional morphological criterion for their identification (inset in Fig. 5A). In 5 dpf embryos, we counted 180 eGFP^+^ cells per embryo including 7-9 cells (5%) that lacked E2Crimson. The proportions of E2Crimson^−^ cells decreased to ∼3% at 7 dpf, 2% at 9 dpf, down to 1-2% in 20 dpf and 30 dpf larva (Fig. 5D). Taking into account the rapid growth of the pancreas during these stages (Wan et al., 2006), this suggests that the total number of *ptf1a^+^*, *ela3l^−^* cells per pancreas remains relatively constant. The data highlight *ptf1a^+^*, *ela3l^−^* cells as a developmentally maintained rare subpopulation within the *ptf1a:eGFP*^+^ pool.

**Fig. 6.**
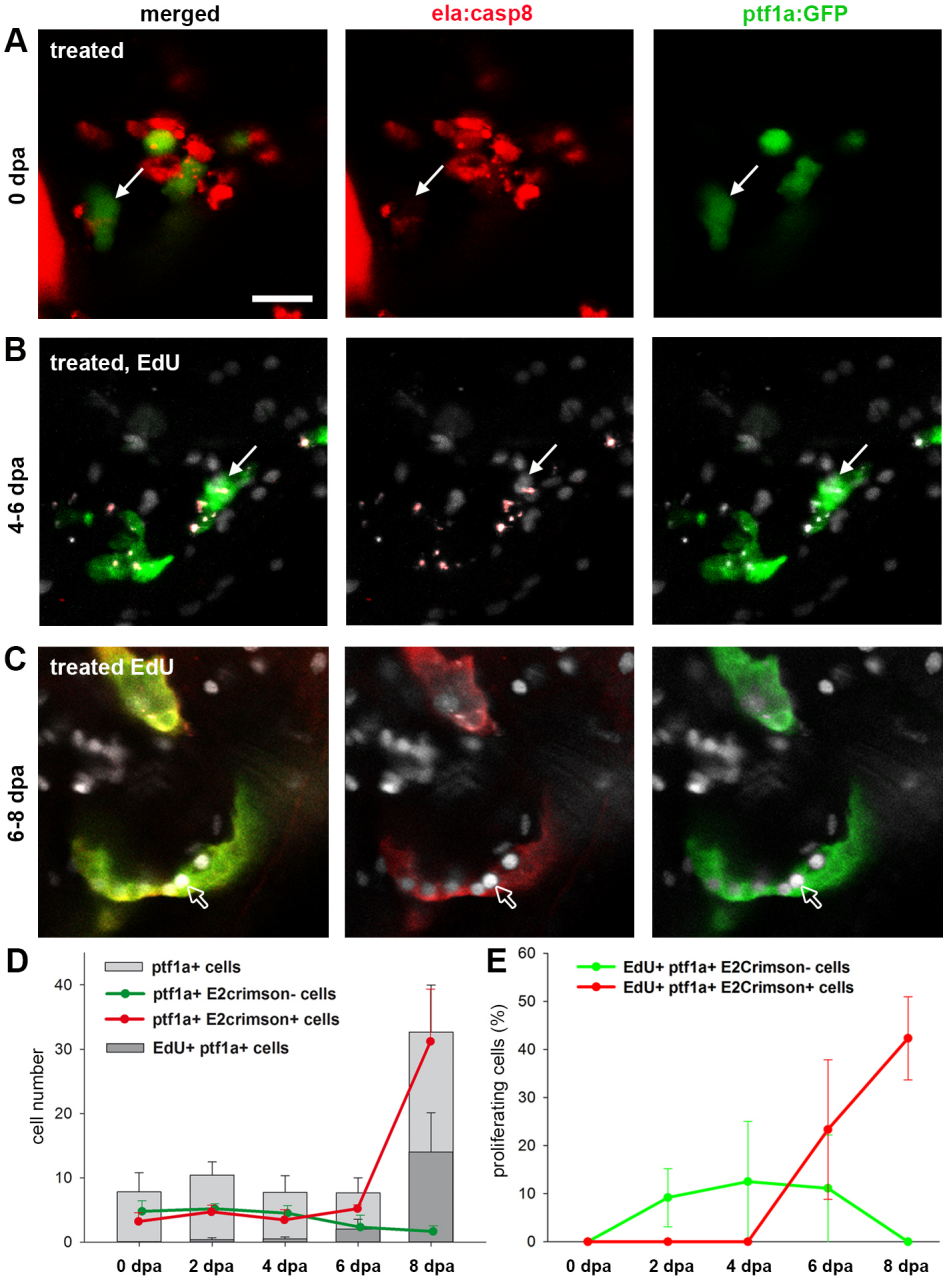
***Ptf1a*^+^ cells as a source for exocrine cell regeneration.** (A-C) Confocal images of the pancreatic head region of EdU-labeled Tg(*ela:casp8; ptf1a:eGFP*) at 0 dpa (A), 6 dpa (B) and 8 dpa (C) following a 7-9 dpf ablation treatment showing that during regeneration, *ptf1a^+^*, *ela3l^−^* cells (white arrows) rarely proliferate and differentiated exocrine cells reconstitute the exocrine pancreas. (D) Absolute numbers of all *ptf1a*^+^ cells in the pancreatic head region, proliferating *ptf1a*^+^ cells and GFP^+^, E2Crimson^−^ and GFP^+^, E2Crimson^+^ cells. (E) Relative amount of EdU^+^, ptf1a^+^, E2Crimson^−^ and EdU^+^, ptf1a^+^, E2Crimson^+^ cells. Mean+s.e.m. Scale bar: 20 µm.

To determine whether and how these cells contribute to exocrine cell recovery we followed regeneration in *ela:casp8;ptf1a:eGFP* larvae over a period of 8 days after a 7-9 dpf ablation procedure. Incubation of larvae in EdU over 2 days before fixation was used to define sites of cell proliferation (Fig. 6). Between 0 and 4 dpa, the pancreatic head region contained around 5-7 eGFP-expressing cells lacking E2Crimson and a similar number of spots with overlapping eGFP and E2Crimson signals (Fig. 6A,D). While these spots were counted as cells, their abnormal morphology (Fig. 6A,B), the absence of *trypsin* expression as revealed by qPCR and *in situ* hybridization analyses (Fig. 1C; Fig. S8) and the complete lack of EdU in a total of 73 cells (Fig. 6E), suggest that most of these signals correspond to apoptotic cells and post-apoptotic cell debris. Starting at 6 dpa, a reduced number of eGFP^+^ E2Crimson^−^ cells could be distinguished, while the number eGFP^+^ E2Crimson^+^ cells rapidly increased and reached around 30 cells/embryo at 8 dpa (Fig. 6D). Consistent with our earlier observation, very few EdU signals were detected in eGFP^+^ E2Crimson^−^ cells (7%, *n*=97 cells), whereas after 6 dpa, more than 32% of eGFP^+^, E2Crimson^+^ cells showed EdU signals (Fig. 6B-E, see also Fig. 4). The data confirm the slowly proliferating *ptf1a^+^*, *ela3l^−^* cells residing in the pancreas as the source for exocrine cell regeneration. They further suggest that concurrent with or after onset of *ela3l* expression at around 6 dpa, these cells switch to rapid proliferation.

As the number of eGFP^+^ E2Crimson^−^ cells appeared to decrease with onset of E2Crimson expression, we were interested to find out if all *ptf1a^+^* cells eventually differentiated into acinar tissue during regeneration. In this case, a second ablation treatment shortly after onset of ela3l expression (10-12 dpa) was expected to cause the complete removal of *ptf1a^+^*, *ela3l^−^* cells. In contrast to this expectation, the corresponding experiment using *ela:casp8* larvae revealed no difference between the number of *ptf1a^+^*, *ela3l^−^* cells after single or after repeated ablation (Fig. S9). In both cases, analyses at 0 dpa revealed about 6-7 *ptf1a^+^*, *ela3l^−^* cells (*n*=7 embryos). This shows that a constant number of *ptf1a^+^*, *ela3l^−^* cells is maintained during regeneration and that only a subpopulation of *ptf1a^+^*, *ela3l^−^* cells contributes to exocrine cell neogenesis.

### Wnt signaling is required for proliferation of exocrine cells during the later regenerative phase

**Fig. 7.**
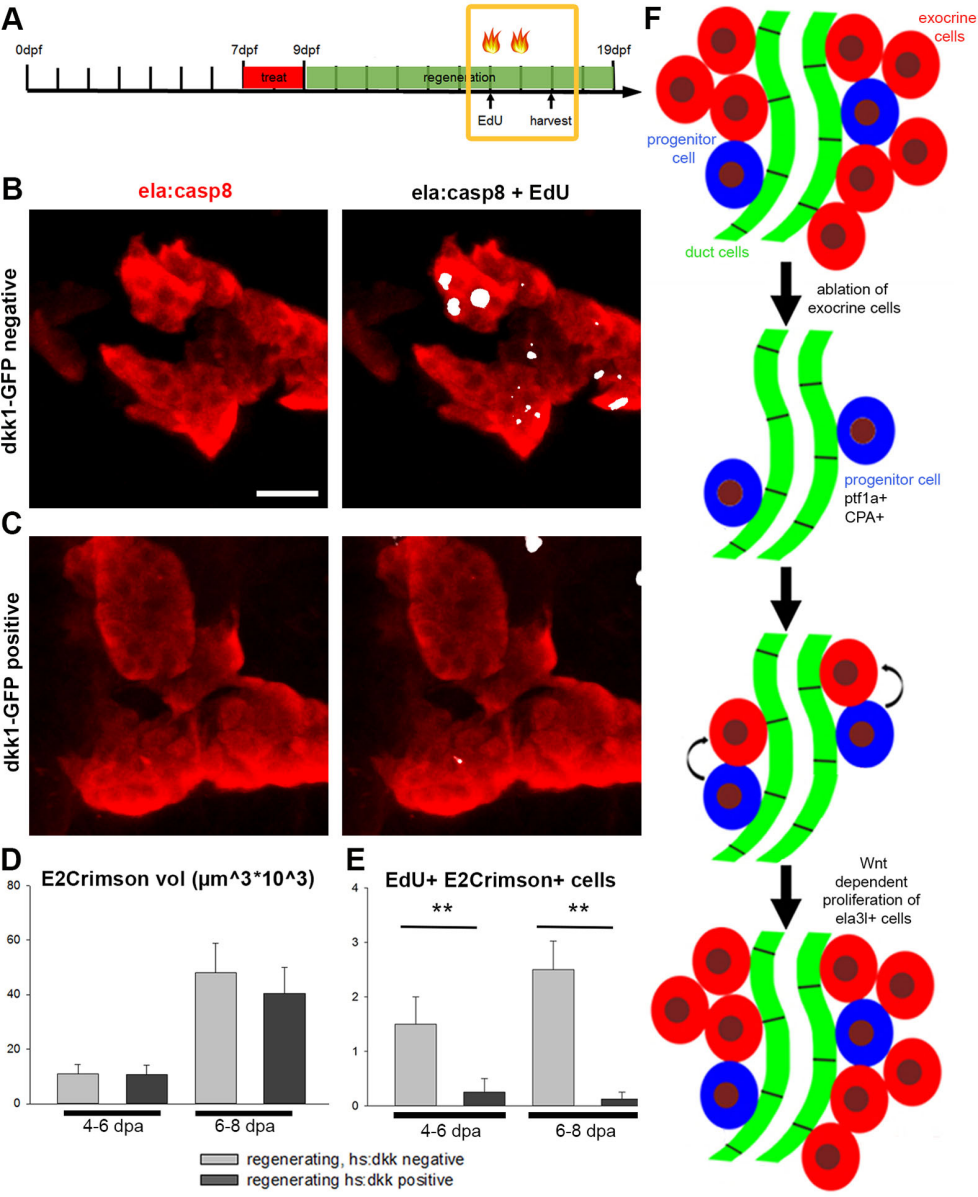
**Proliferation of exocrine cells during regeneration is dependent on Wnt signaling.** Confocal projections of Tg(*ela:casp8*) crossed to Tg(*hsp70l:dkk1-eGFP*) and treated with 5 µM Dim. During regeneration (4-6 dpa or 6-8 dpa) larvae were heat shocked twice to induce *dkk1* and they were additionally injected with EdU. (A) Timeline indicating treatments and heat shock (flames). (B) Control littermate negative for *dkk1-eGFP*. (C) Larvae expressing *dkk1-eGFP* showed reduced numbers of EdU^+^ cells. Volume of E2Crimson cells (D) and EdU^+^ E2Crimson^+^ cells (E) in larvae expressing *dkk1-eGFP* and controls at 6 and 8 dpa during regeneration, showing that the induced expression of *dkk1* reduces the number of EdU^+^ exocrine cells (*N*>4 larva for each time point). Mean+ s.e.m.; **P<0.001. (F) Model of exocrine cell regeneration after complete removal of exocrine cells. Scale bar: 20 µm.

Canonical Wnt signaling has been reported to play a key role in exocrine cell proliferation and regeneration in mouse (Keefe et al., 2012; Murtaugh et al., 2005; Murtaugh and Keefe, 2015; Nakhai et al., 2008; Wells et al., 2007). To test whether Wnt signaling has similar functions in zebrafish, we used the Tg(*hsp70l:dkk1-eGFP*) line for conditional activation of the Wnt-signaling inhibitor dickkopf 1 (Dkk1) (Stoick-Cooper et al., 2007). Consistent with a conserved requirement for Wnt signaling in normal late pancreatic development, in *hsp70l:dkk1-eGFP* embryos that received two heat shocks at 5 and 6 dpf, *ela3l* expression was reduced by 26% at 7 dpf (Fig. S10). To determine the functions of Wnt during regeneration, *ela:casp8;hsp70l:dkk1-eGFP* animals were treated with Dim at 7 to 9 dpf and then heat shocked twice either at 4 and 5 dpa or at 6 and 7 dpa (Fig. 7A). EdU treatment started 1 hour after the first heat shock and continued until fixation 1 day after the second heat shock; these were used as a quantitative measure of mitosis (Fig. 7B-D). While induction of *dkk1* had only mild effects on the volume of exocrine tissue, we observed significantly reduced numbers of EdU^+^ cells in *dkk1*-induced embryos compared with sibling larvae lacking the *hsp70l:dkk1-eGFP* transgene. The number of EdU^+^ E2Crimson^+^ cells per pancreatic head region was reduced by more than 83% in animals heat treated at 4 dpf and 5 dpf, and by 95% in animals heat treated at 6 and 7 dpf (Fig. 7B,C). In conclusion, this suggests that proliferation of exocrine cells during development and regeneration is dependent on Wnt signaling.

## DISCUSSION

In this study we used the zebrafish as a model system to study exocrine pancreas regeneration. As part of these experiments we introduced two genetically inducible cell ablation systems into the zebrafish and we demonstrate that both systems allow highly efficient and specific removal of exocrine tissue in larvae and adult animals. Furthermore, we show that zebrafish larvae are able to recover from a complete loss of exocrine tissue in less than 2 weeks, and we identified a developmentally maintained rare population of *ptf1a^+^*, *ela3l^−^* cells as the source of this regeneration. Our data suggest that exocrine regeneration requires an initial proliferation-independent maturation process in these cells, which is then followed by a phase of Wnt-regulated rapid proliferation and cell expansion.

### Introduction of two alternative methods for genetically encoded inducible cell ablation to zebrafish

The zebrafish embodies a remarkable regenerative capacity as it is able to regenerate liver (Choi et al., 2014), endocrine β-cells in embryonic and adult stages (Curado et al., 2007; Delaspre et al., 2015; Ghaye et al., 2015; Moss et al., 2009; Pisharath et al., 2007), heart, neurons and even whole fins, including bone and cartilage tissue (reviewed in Gemberling et al., 2013). Conditional targeted ablation is a powerful tool to study the role of specific cell lineages, tissues or physiological responses involved in regenerative processes. Several genetically encoded systems have been tested transiently in zebrafish embryos (reviewed in Curado et al., 2008), but until now, the only efficient and reliable method was the NTR-mediated conversion of antibiotic metronidazole into a cytotoxic agent (Curado et al., 2007; Pisharath et al., 2007). Here, we introduce and validate two alternative approaches for inducible, genetically encoded cell ablation in zebrafish. We show that both approaches are highly efficient in removing cells of the exocrine compartment of the pancreas without affecting adjacent tissues. Treatment of embryos expressing FKBP-caspase-8 with Dim induced apoptosis of cells within 8 h and resulted in >95% reduced expression of exocrine markers within 48 h. The DT-mediated approach was similarly efficient but slower, probably since it is not directly interacting with the cellular apoptosis machinery (Morimoto and Bonavida, 1992). In this approach, the first apoptotic cells appeared after 60 h of treatment, and a virtually complete removal of cells was achieved between 72 and 96 h. Furthermore, both methods were also highly efficient and specific in ablating exocrine tissue in adult animals and in removing the insulin-producing β-cells of the endocrine pancreas. Dim-mediated ablation as well as DT-induced removal of cells in adult animals was achieved after a single injection within a few days, as revealed by the loss of fluorescent cells and by the lack of these cells in histological sections. Regeneration models using juvenile or adult animals rather than embryos become more important as they better resemble human pathologies. Furthermore, regeneration events in embryonic stages potentially represent a continuation of developmental events and might not be the same as regeneration in adult animals (Hesselson et al., 2009). While additional experiments will be required for a detailed evaluation and comparison of potentially missed side effects, our results highlight both ablation approaches as an attractive alternative to NTR/Met for future ablation studies using zebrafish. Unlike NTR/Met, both methods utilize eukaryote-specific pathways for cell ablation that should not interfere with microorganisms. Therefore, these methods might be of particular interest for studying the role of internal microbiota, which have recently gained major attention as important modulators of physiology, disease and regeneration (Bates et al., 2006; Chang and Lin, 2016; Cheesman et al., 2011; Hill et al., 2016; Kostic et al., 2013; Reikvam et al., 2011; Shanahan, 2013).

### Distinct modes of exocrine cell regeneration

Regeneration studies have revealed an amazing flexibility and plasticity in tissue recovery. For example, observations of β-cells and liver regeneration showed that different sources for newly forming cells can be mobilized depending on the extent, timing and cell-specificity of tissue disruption (Aloia et al., 2016; Chera and Herrera, 2016; Kopp et al., 2016). Different modes of regeneration have also been described for exocrine tissue recovery in mouse. Following partial ablation by caerulein treatment or duct ligation, the exocrine tissue was re-established primarily by proliferation of residual exocrine cells (Desai et al., 2007; Strobel et al., 2007). Another study using genetically encoded expression of the human DTR in exocrine cells and administration of DT induced almost complete ablation of exocrine cells and showed regeneration by differentiation from the mature duct, terminal duct cells, centroacinar cells or another unidentified progenitor cell population (Criscimanna et al., 2011). While our data suggest a different source for the regenerating acinar cells in exocrine tissue-depleted zebrafish larvae, they also revealed two distinct modes or phases of regeneration. In particular, we find that the complete removal of *ela3l^+^* cells resulted in a phase of about 4 days with minor pancreatic proliferation and no exocrine maturation, which was then followed by onset of *ela3l* expression and massive proliferation of *ela3l^+^* cells. These results are consistent with a two-phase regeneration process, with phase 1 being the slow neogenesis of *ela3l^+^* cells, and phase two, the rapid proliferation of mature or partly matured *ela3l^+^* cells as the major mechanism of exocrine tissue recovery. In this context, it is interesting to note that the faster recovery of exocrine tissue after treatment at 5-7 dpf compared with 7-9 dpf correlates with the appearance of new proliferating *ela3l^+^* cells shortly after the earlier ablation. These cells most likely escaped ablation because of the later onset of *ela3l* expression as part of the normal differentiation process.

### *ptf1a^+^*, *ela3l^−^* cells are a novel source of regenerating exocrine cells in zebrafish larvae

Here, we identified a developmentally maintained rare subpopulation of *ptf1a^+^*, *ela3l^−^* cells and we propose these cells as the source of newly forming exocrine cells after complete removal of acinar cells. These cells were positive for CPA, and they showed no signs of zymogen granules, consistent with the lack of ela3l:E2Crimson signal. As CPA and Ptf1a expression have been reported to precede that of zymogens such as elastase in normal exocrine maturation (Gittes and Rutter, 1992; Guerrera et al., 2015; Han et al., 1986; Schick et al., 1984), these cells might represent ordinary differentiating exocrine cells not yet (or weakly) expressing *ela3l*. While our data do not entirely exclude this option, the time frame of differentiation and the maintenance of these cells during larval stages and after repeated ablation argue against it. Based on the reported delay of 1-2 days between CPA and *ela3l* expression, a maturation-dependent recovery of *ela3l* should start immediately after ablation, as it was only the case after 5-7 dpf ablation treatments not after later ablation (Yee et al., 2005). Our data suggest *ptf1a^+^*, *ela3l^−^* cells as a previously unrecognized progenitor population for acinar cell neogenesis. These cells could be monopotent progenitor cells similar to the STMN1^+^ cells recently identified in the adult mouse pancreas (Wollny et al., 2016). While STMN1^+^ and *ptf1a^+^*, *ela3l^−^* cells both share a low prevalence in the mature pancreas, STMN1^+^ cells, unlike the *ptf1a^+^*, *ela3l^−^* cells, express mature acinar markers such as trypsin and elastase. Alternatively, the *ptf1a^+^*, CPA^+^ cells might represent residual multipotent potential progenitor cells with the potency to form endocrine, exocrine and duct cells, which have been reported during the early phase of mouse pancreas development (Zhou et al., 2007). Recently, lineage-tracing studies in zebrafish revealed the presence of a similar multipotent progenitor population in early pancreatic development (Wang et al., 2015). Using a *ptf1a:CreER^T2^* line, the contribution of early stage *ptf1a*^+^ cells to ∼6% of NRCs in 6 dpf embryos and to adult endocrine cells was shown. Corresponding mouse lineage-tracing studies using *Cpa1^CreERT2^* and *Ptf1a^CreERTM^* also revealed a shift from multipotency to acinar-specific unipotency after secondary transition (Zhou et al., 2007), while multipotency was found to be (re)activated in Ptf1a^+^ cells by injury, e.g. partial duct ligation (Pan et al., 2013). Interestingly, earlier regeneration studies using *Elastase^CreERT2^* mice for cell tracking indicated an acinar-restricted differentiation potential in pre-existing acinar cells (Desai et al*.,* 2007). It had been suggested that the lower *Cre/loxP* labeling efficiency of *Elastase^CreERT2^* compared with *Ptf1a^CreERTM^* might have hindered detection of the rare endocrine contributions in the *Elastase^CreERT2^* experiments (Pan et al., 2013). Alternatively, *Ptf1a^CreERTM^* might label additional cell populations and the potential for multipotency could be restricted to an Elastase^−^, Ptf1a^+^ subpopulation. While future studies using genetic tracing approaches in combination with tissue-specific or *ptf1a*-regulated cell ablation will be required to clarify this option, it is tempting to speculate that *ptf1a^+^*, *ela3l^−^* cells resemble a conserved multipotent pancreatic progenitor population.

### Maintenance of *ptf1a^+^*, *ela3l^−^* cells during development and regeneration

We find that the proportion of *ptf1a^+^*, *ela3l^−^* cells decreases with age, whereas the total number appears to be unaffected by age and even by repeated acinar ablation. This highlights *ptf1a^+^*, *ela3l^−^* cells as a maintained cell population of the pancreas. Currently, we can only speculate about the molecular and cellular mechanism regulating their maintenance, proliferation or induction of acinar differentiation. The constant number of *ptf1a^+^*, *ela3l^−^* cells during regeneration suggests asymmetric cell division with only one daughter cell initiating *ela3l* expression as the likely mechanism for progenitor maintenance. While this type of proliferation normally depends on lateral inhibition through Notch signaling, our short-term NRC-tracking analyses, in agreement with genetic lineage-tracing studies (Wang et al., 2011, 2015), excluded an involvement of canonical Notch signaling in exocrine regeneration. Interestingly, the lack of Notch responsiveness in acinar cells contrasts with the various requirements for Notch signaling and individual Notch signaling mediators in exocrine differentiation and regeneration (Esni et al., 2004; Hidalgo-Sastre et al., 2016; Kopinke et al., 2012; Siveke et al., 2008; Yee et al., 2005; Zecchin et al., 2007). Activation of the TP1-Notch reporter requires interaction of the intracellular part of Notch receptors (NICD) with its cofactor RBP-Jκ (Parsons et al., 2009). As NICD and Pft1a directly compete for RBP-J binding (Beres et al., 2006; Masui et al., 2007), Notch signaling might regulate *ptf1a^+^*, *ela3l^−^* fates without inducing TP1 reporter by reducing interactions between Ptf1a and RBP-J (Esni et al., 2004; Gradwohl et al., 2000; Heremans et al., 2002). Consistent with this option, Ptf1a levels had been found to be crucial for the differentiation potential of *pft1a*-expressing cells (Wang et al., 2015).

### Proliferation of *ela3l^+^* cells and requirement of Wnt signaling in exocrine recovery

The 4- to 5-fold increase of *ela3l^+^* cell numbers between 4 and 6 dpa (Figs 3 and 6) demonstrate an amazing proliferation capacity of these cells, particularly as not all cells appear to contribute to acinar cell recovery. The lack of EdU signals in more than 50% of *ela3l^+^* cells suggests that only a subset of these cells is proliferating with a rate above 1 division per day. In agreement with the rare division events observed in *ptf1a^+^*, *ela3l^−^* cells, this further suggests that very low numbers of newly formed *ela3l^+^* cells are sufficient to explain the observed recovery dynamics. In this context, the accumulation of newly forming *ela3l^+^* cell in a few cell clusters of 8 dpa animals (for example, 3 clusters with 6-32 cells in Fig. 3H′″) is consistent with the formation of these clusters from individual *ela3l^+^* cells. The heterogeneity in the proliferation behavior among the *ela3l^+^* cells reveals a rapid functional diversification. In analogy to adult stem cell systems, the newly formed *ela3l^+^* cells might first expand as transit-amplifying cells before maturation into non-proliferating acinar cells. However, the striking similarities to the recently described heterogeneity among adult mouse acinar tissue (Wollny et al., 2016) support an alternative explanation for the different proliferation behavior of *ela3l*^+^. Accordingly, newly formed *ela3l^+^* cells could be mono-potent acinar-specific progenitors with long-term self-renewal capacity.

Finally, we found that proliferation of newly generated exocrine cells was strongly impaired upon induction of the Wnt antagonist Dkk1. As mouse studies previously established canonical Wnt signaling as a key regulator of differentiation, proliferation, maintenance and regeneration of acinar tissue (Keefe et al., 2012; Morris et al., 2010; Murtaugh et al., 2005; Murtaugh and Keefe, 2015; Nakhai et al., 2008; Wells et al., 2007), our results are consistent with a conserved requirement for Wnt signaling in acinar cell proliferation.

In early zebrafish development, Wnt signaling has been associated with hepatic specification and proliferation, and the inhibition of pancreatic specification (Goessling et al., 2008; Poulain and Ober, 2011). More recently, it was shown that prostaglandin E2 has the same effects in early development as activated Wnt signaling, but when applied at later stages, it also promotes expansion of the exocrine pancreas (Nissim et al., 2014). Considering the genetic interaction of prostaglandin E2 and Wnt signaling (Goessling et al., 2009), this suggested a conserved connection with Wnt signaling and late embryonic expansion of exocrine tissue. Consistent with this notion and in agreement with Wnt-specific interference, we found that induction of *dkk1* reduced expression of the liver and acinar marker. Opposing effects of Wnt signaling on preventing early pancreas specification and supporting exocrine expansion have also been shown in mouse, where an early upregulation of β-catenin prevents normal formation of exocrine and endocrine compartments whereas upregulation at a later time point causes enhanced proliferation and increase in pancreas size (Heiser et al., 2006). Interestingly, the volume of E2Crimson^+^ regenerated tissue was not reduced in the *dkk1*-induced animal as would be expected. Possibly, the short time window after heat treatment was sufficient for preventing S-phase-dependent EdU incorporation in the *dkk1-GFP*-induced embryo, but not long enough to interfere with a full division cycle. As increased Wnt activity resulted in smaller acinar cells (Heiser et al., 2006), the unchanged acinar volume might also result from interference with exocrine cell size.

### Conclusions

We introduced two alternative approaches enabling complete ablation of pancreatic cells in the zebrafish and by using these techniques, we found *ptf1a^+^* cells not expressing *ela3l* as a novel source of pancreatic regeneration. Furthermore, we identify Wnt signaling as important for the expansion of exocrine cell mass. Our data suggest a two-phase regeneration process following severe loss of acinar cells (Fig. 7F). In phase one, the loss of acinar cells activates a regeneration program in a subset of *ptf1a^+^*, *ela3^−^* cells. As the number of *ptf1a^+^*, *ela3^−^* cells did not change during regeneration, this program must include mechanisms to coordinate maintenance of *ptf1a^+^*, *ela3^−^* cells and initiation of acinar differentiation. Once *ela3l* expression is re-established, these cells in phase 2 undergo rapid Wnt-dependent cell division, leading to a fast recovery of *ela3l^+^* tissue. While we cannot completely exclude the possibility that other potential progenitor sources contribute to the regeneration, it is tempting to conclude that these cells represent stem cells able to restore exocrine cells. Therefore, it will be interesting to study regenerative processes in *ptf1a*-depleted juvenile and adult fish to determine possible different sources and mechanisms. Further studies addressing the expression profile of these rare cells, detailed clonal fate analyses using genetic labeling approaches and sequential treatment with different thymidine analogs are required to characterize them and their full progenitor potential.

## MATERIALS AND METHODS

### Cloning of transgenic constructs

Transgenic lines were generated using the ‘Tol2-Kit’ (Kwan et al., 2007). 1.9 kb of the *elastase3l* promoter (Wan et al., 2006) and 1 kb of the *insulin* promoter (provided by Francesco Argenton, University of Padova, Italy) were cloned into the p5E-MCS. The DTR constructs were prepared by cloning the human *HB-EGF(I117V/L148V)* cDNA (Furukawa et al., 2006) into p5E-ela3l and the p5E-ins plasmids. The *FKBP-caspase8* fusion cDNA (Pajvani et al., 2005) (a gift from Philipp Scherer, University of Texas Southwestern Medical Center, USA) was also cloned downstream into the p5E-ela3l and p5e-ins plasmids. E2Crimson (pE2-Crimson-N1 Vector, 632554, Clontech) under the control of *ela3l* or *insulin* promoters were cloned into the pME-MCS plasmid. For the *ela3l:H2B-eGFP* construct *H2B-eGFP* cDNA (obtained from Joachim Wittbrodt, University of Heidelberg, Germany) was cloned in the pME-MCS plasmid. Constructs were fused together by the LR recombination reaction as described (Kwan et al., 2007) in the pDestTol2pA to generate Tg(*ela3l:DTR;ela3l:E2Crimson*), abbreviated to *ela:DTR*, Tg(*ins:DTR;ins:E2Crimson*), abbreviated *ins:DTR*, Tg(*ela3l:caspase8;ela3l:E2Crimson*), abbreviated to *ela:casp8* and Tg(*ins:caspase8;ins:E2Crimson*), abbreviated to *ins:casp8*. Corresponding DNAs were injected with transposase mRNA into fertilized eggs to generate transgenic fish.

### Zebrafish maintenance and fish lines

Zebrafish (*Danio rerio*) were maintained according to standard protocols. Most lines are kept in the *Mitfa^b692/b692^/ednrb1^b140/b140^* background (a gift from Wolfgang Driever, University of Freiburg, Germany).

Additional lines used in this report are: Tg(*Tp1:eEGFP*) (Parsons et al., 2009) and Tg(*Tp1:H2B-mcherry*) (Ninov et al., 2012), Tg(*cld:lyn-eEGFP*) (Haas and Gilmour, 2006), Tg(*gcga:eGFP*) (Zecchin et al., 2007), Tg(*nkx2.2a:eGFP*) (Pauls et al., 2007), Tg(*ptf1a:eGFP*) (Park et al., 2008), Tg(*hsp70l:dkk-eGFP*) (Stoick-Cooper et al., 2007). Heat shocks for *dkk1* induction were performed at 39°C for 60 min.

### Drug dependent cell ablation

Diphtheria toxin (DT, D0564, Sigma) was reconstituted in sterile distilled water to 1 mg/ml and diluted to the indicated concentrations in eggwater. AP20187 (dimerizer, Dim) is supplied at 500 µM in 100% ethanol (635059, Clontech) and this stock was directly diluted in eggwater. Control embryos were exposed to corresponding volumes of ethanol. Treated embryos were incubated at 28°C in the dark. F2 transgenic embryos were used to study the dose response of DT and Dim in ablating the exocrine pancreas. At 5 dpf, embryos were incubated in 10 and 15 µg/ml DT and 1.6-8 µM Dim. Adult *ela:DTR* and *ela:casp8* zebrafish were injected intraperitoneally with 20 ng/g DT and 75 ng/g Dim (both diluted in 5 mmol/l citrate, pH 5) and loss of fluorescence was monitored via epifluorescence microscopy.

### Whole-mount TUNEL staining

Embryos were fixed for 1-2 h in 4% paraformaldehyde (PFA), 1% DMSO and permeabilized by incubation in 1×PBS, 0.2% Triton X-100, heads and tails were cut off and the gut was cut open. After proteinase K digestion (10 µg/ml) for 15 min and re-fixation in 4% PFA for 20 min at room temperature, staining was performed using the *In situ* Cell Death Detection Kit, Fluorescein (Roche, 11684795910) according to the manufacturer's instructions.

### EdU staining

For EdU-based detection of proliferating cells, the Click-IT 647 Kit (Invitrogen, C10085) was used. In all experiments, animals were incubated in EdU for 48 h before fixation. Treatments were started with an injection of 5 nl EdU solution (100 µM EdU, 2% DMSO) into the common cardinal vein (5-11 dpf) (Kramer-Zucker et al., 2005) and at later stages (>11 dpf) in the gut of anaesthetized animals (0.6 mM tricaine). Subsequently, the injected fish were incubated in 50 µM EdU, kept in the dark for 48 h and then harvested. Samples were fixed in 4% PFA for 1-2 h at RT. After three washes in 1×PBS, 0.2% Triton X-100, heads and tails were removed and the gut was cut open. EdU staining was performed according to manufacturer's instructions.

### Immunofluorescence

Embryos were fixed for 1-2 h at room temperature in 4% PFA, washed for 3×5 min with 1×PBS, 0.2% Triton X-100, head and tail were cut off and the gut was cut open. Embryos were incubated in blocking buffer containing 1% DMSO, 1% sheep serum, 1% BSA and 1% Triton X-100 in 1×PBS for at least 60 min at room temperature. The embryos were then incubated overnight at 4°C with primary and secondary antibodies, using 1:200 and 1:1000 dilutions, respectively. Primary antibodies: rabbit anti-carboxypeptidase A/CPA (Chemicon, AB1213), mouse anti-eGFP (Roche, 11814460001), rabbit anti-dsRed (Clontech, 632496). Alexa Fluor 488 and Alexa Fluor 546 conjugated anti-rabbit and anti-mouse were used as secondary antibodies (Invitrogen, A11001, A11010).

### Whole-mount *in situ* hybridization

*In situ* hybridization was performed with digoxigenin-labeled antisense RNA probes (DIG RNA Labelling Mix, Roche) and anti-digoxigenin-AP antibody (1:4000, Roche) using previously published protocols (Hauptmann and Gerster, 2000). The *trypsin* antisense RNA probe [obtained from Francesco Argenton (Biemar et al., 2001)] was generated after linearizing the corresponding plasmid and using Sp6 RNA polymerase.

### RT-qPCR

Total RNA samples were prepared from pools of 5-10 embryos or larvae at indicated time points using Trizol Reagent (Ambion). cDNA was prepared using the Maxima First Strand cDNA Synthesis Kit (Thermo Fisher Scientific, K1641). HOT FirePol EvaGreen qPCR Mix Plus (Solis BioDyne, 08-24-00020) was used for qPCR reactions in a CFX Connect Real-Time System (Bio-Rad). Reactions were performed on at least two biological samples with two technical replicates each. Primers were designed using ‘QuantPrime’ (Arvidsson et al., 2008) as follows (5′-3′ orientation): *β-actin*: F, CTGCTCTGTATGGCGCATTGAC and R, GTTAGACAACTACCTCCCTTTGCC; *ela3l*: F, GTTGTCGCTGGATGCAATGGAG and R, TGCCGTCAGAGTTCTTGCAGTTC; *trypsin*: F, ACCAGCTGTCTGATCTCTGGATGG and R, CAGACGGCTTGGGTAATTGCTTC; *dkk1b*: F, TGCACATCGCCATGCTCTCTAC and R, TAGAACCGGCCACTTTGATGCAG; *fabp10a*: F, TGGACGGCAAGAAGCTCAAGTG and R, GGTCAGTTCTGCAGACCAGCTTTC; *rpl13αI*: F, ACAGGCTGAAGGTGTTTGATGGC and R, GGACAACCATGCGCTTTCTCTTG; *loopern4*: F, TGAGCTGAAACTTTACAGACACAT and R, AGACTTTGGTGTCTCCAGAATG; *ins*: F, GCCCAACAGGCTTCTTCTACAAC and R, GCAGATTTAGGAGGAAGGAAACCC.

### Histological analysis

Inner organs of adult fish were fixed in 4% PFA overnight at 4°C, then specimens were serially dehydrated with ethanol solutions and embedded in paraplast wax. Serial sections for histological studies were 8 µm thick and stained with Hematoxylin and Eosin (H&E).

### Image acquisition

Confocal images were generated as previously described (Arkhipova et al., 2012) with a Zeiss LSM Exciter5 microscope equipped with 25 mW Argon laser (468, 488 and 514 nm), 1 mW 543 nm He-Ne laser, and a 5 mW 633 nm He-Ne laser. Fluorescence detection was performed with the following filters: BP 505-550 (eGFP and Alexa 488), LP 560 (E2Crimson, mCherry) and LP 650 (Alexa 633, Click-IT 647). Additional confocal images were generated with a Leica TCS SP5 II Laser using 488 nm and 561 nm excitation wavelengths. Fluorescence detection was performed a bandwidth PMT 500 to 550 nm and a Hyd 3 bandwidth 600 to 650 nm. Adobe Photoshop CS5 was used for image arrangement and adjustment. Epifluorescence images were taken at the Leica MZ16A stereomicroscope using either a Leica DFC320 camera or a Zeiss AxioCam ICm1 camera.

### Statistics

Volumetric analyses of E2crimson- and eGFP-expressing tissue and automatic cell counting of *ptf1a:eGFP*^+^, EdU^+^ cells and *ela3l:H2B-GFP*^+^ cells were performed using Imaris software (v.7.3, Bitplane). Endocrine cells marked by GFP (*ins:CD4-GFP*) and E2Crimson expression (*ins:casp8*, *ins:DTR*) were counted using the Cell Counter plugin of ImageJ (http://rsbweb.nih.gov/ij/plugins/cell-counter.html). Statistical analysis and graphs were performed with SigmaPlot (v.12.5, Systat Software) using Student's *t*-test or one-way analysis of variance (ANOVA) and pairwise multiple comparison using the Holm–Šídák method. *P*-values for statistical significance are stated. All graphs show mean+s.e.m.
